# Correction: Chrysomycins A–C, antileukemic naphthocoumarins from *Streptomyces sporoverrucosus*

**DOI:** 10.1039/d2ra90078e

**Published:** 2022-08-19

**Authors:** Shreyans K. Jain, Anup S. Pathania, Rajinder Parshad, Chandji Raina, Asif Ali, Ajai P. Gupta, Manoj Kushwaha, Subrayashastry Aravinda, Shashi Bhushan, Sandip B. Bharate, Ram A. Vishwakarma

**Affiliations:** Natural Products Chemistry Division, Indian Institute of Integrative Medicine (CSIR) Canal Road Jammu 180001 India ram@iiim.ac.in +91-191-2569333 +91-191-2569111; Academy of Scientific & Innovative Research (AcSIR) Anusandhan Bhawan, 2 Rafi Marg New Delhi 110001 India; Cancer Pharmacology Division, Indian Institute of Integrative Medicine (CSIR) Canal Road Jammu 180001 India; Fermentation Division, Indian Institute of Integrative Medicine (CSIR) Canal Road Jammu 180001 India; Quality Control and Quality Assurance Division, Indian Institute of Integrative Medicine (CSIR) Canal Road Jammu 180001 India; Medicinal Chemistry Division, Indian Institute of Integrative Medicine (CSIR) Canal Road Jammu 180001 India sbharate@iiim.ac.in

## Abstract

Correction for ‘Chrysomycins A–C, antileukemic naphthocoumarins from *Streptomyces sporoverrucosus*’ by Shreyans K. Jain *et al.*, *RSC Adv.*, 2013, **3**, 21046–21053, https://doi.org/10.1039/c3ra42884b.

The authors regret that incorrect versions of Fig. 6 and Fig. 7 were included in the original article. The correct versions of Fig. 6 and 7 are presented below.

**Fig. 6:**
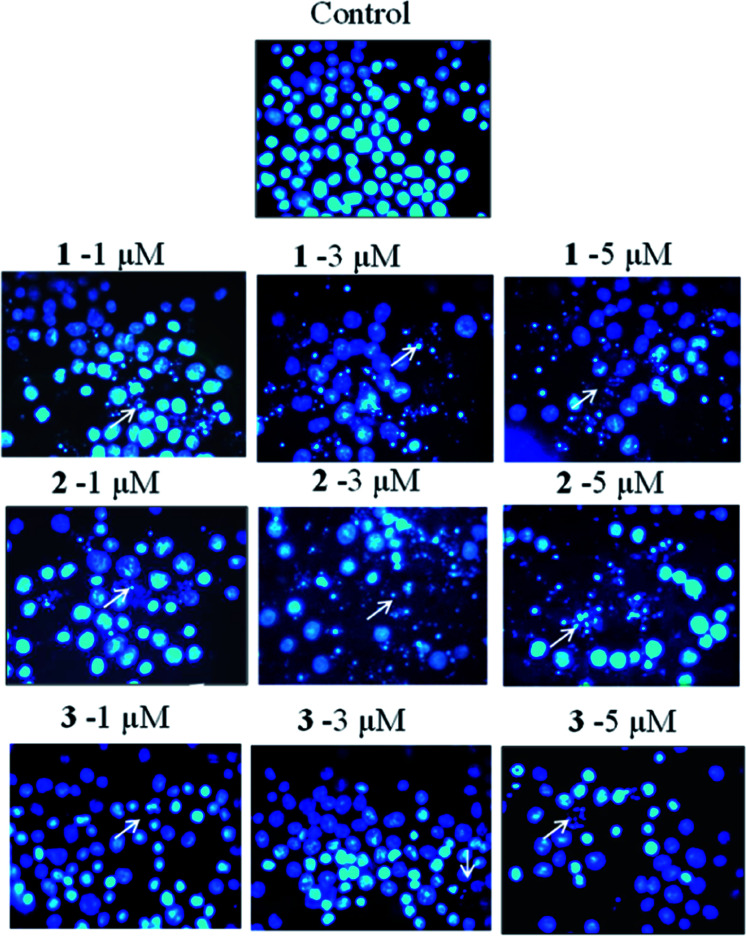
Influence of compounds **1–3** on the nuclear morphology of human leukaemia HL-60 cells. The cells were treated with 1, 3 and 5 μM concentrations of these compounds for 24 h and stained with Hoechst 33258 for 40 min. The altered nuclear morphology and apoptotic bodies indicated by white arrows are seen in treated cells while the nuclei of the untreated cells were round and intact.

**Fig. 7:**
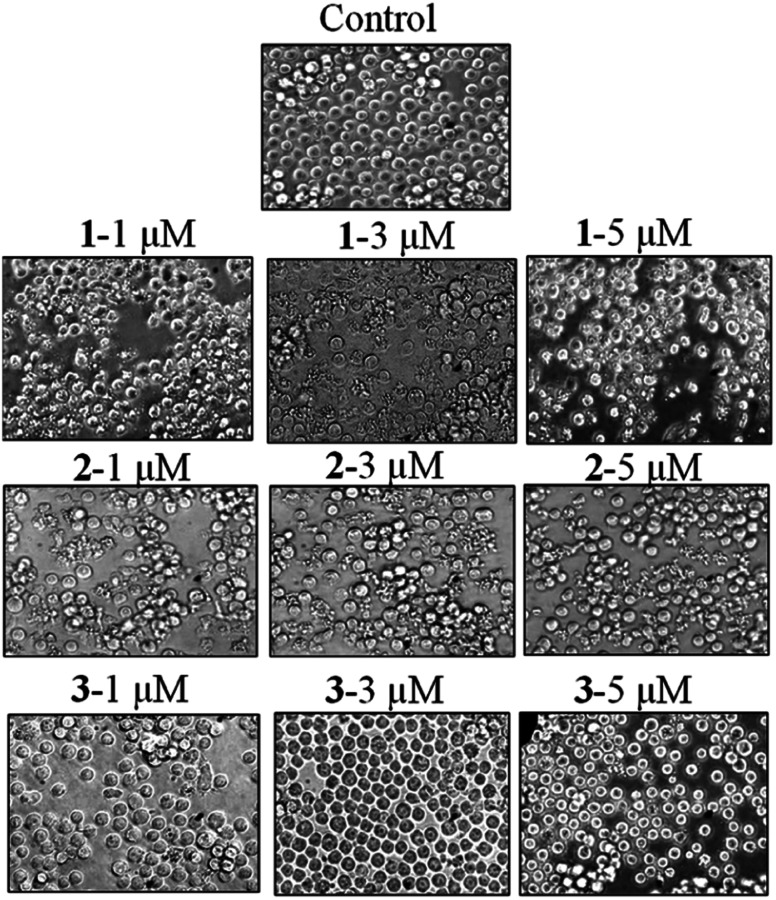
Phase contrast microscopy of compound-treated leukaemia HL-60 cells. Cells were treated with compounds **1–3** at 1, 3 and 5 μM for 24 h and visualized using a phase contrast microscope (Olympus1X72). The morphology of treated cells altered in a concentration-dependent manner, while the untreated cells remained healthy.

The Royal Society of Chemistry apologises for these errors and any consequent inconvenience to authors and readers.

## Supplementary Material

